# Development of Chinese mental health first aid guidelines for psychosis: a Delphi expert consensus study

**DOI:** 10.1186/s12888-020-02840-5

**Published:** 2020-09-10

**Authors:** Wenjing Li, Anthony F. Jorm, Yan Wang, Shurong Lu, Yanling He, Nicola Reavley

**Affiliations:** 1 Centre for Mental Health, Melbourne School of Population and Global Health, University of Melbourne, Level 4, 207 Bouverie Street, Carlton, VIC 3053 Australia; 2 Shanghai Mental Health Centre, Shanghai, China; 3 The Nossal Institute for Global Health, Melbourne School of Population and Global Health, University of Melbourne, Melbourne, VIC Australia

**Keywords:** Mental health first aid, Psychosis, Delphi study, Mainland China

## Abstract

**Background:**

Family and friends of a person developing a mental illness or in a mental health crisis can help the person until treatment is received or the crisis resolves. Guidelines for providing this ‘mental health first aid’ have been developed and disseminated in high-income countries. However, they may not be appropriate for use in China due to cultural and health care system differences. The aim of this study was to use the Delphi expert consensus method to develop culturally appropriate guidelines for a member of the public providing mental health first aid to someone with psychosis in mainland China.

**Methods:**

A Chinese-language survey, comprising statements about how to provide mental health first aid to a person with psychosis, was developed. This was based on the endorsed items from the first round of the English-language questionnaire for high-income countries. These statements were rated by two expert panels from mainland China – a mental health professional panel (*N* = 31) and a lived experience panel (*N* = 41) – on how important they believed each statement was for a member of the public providing first aid to a person with psychosis in China. There were three Delphi rounds, with experts able to suggest additional items in Round 1. Items had to have at least 80% endorsement from both panels for inclusion.

**Results:**

Out of 208 statements, 207 were endorsed for inclusion in the Chinese-language guidelines. Eight new statements were also included. Compared to the English-language guidelines, the importance of family involvement was emphasized in the development of the Chinese-language guidelines.

**Conclusions:**

While many of the actions in the English-language guidelines were endorsed by Chinese participants, a number of additional items point to the importance of developing culturally appropriate mental health first aid guidelines. These guidelines will form the basis for the development of Chinese Mental Health First Aid course aiming at training members of the public on how to provide first aid to someone with a mental health problem.

## Background

A large-scale epidemiological study, covering four provinces of China, found that 27.6% of those diagnosed with psychotic disorders had not sought any type of professional help [1]. Indeed, in comparison with Western countries, duration of untreated psychosis (DUP) has been found to be longer in mainland China [2–4]. Importantly, untreated psychosis and delayed treatment have been demonstrated to have a significant impact on a person’s functional capacity, with a longer DUP predicting poorer cognitive and social functioning (e.g., attention, memory and verbal learning) [5–8]. The worsening of symptoms and functional decline can also result in decreased quality of life in both people with psychosis and their caregivers [9, 10].

One of the most important barriers to seeking professional mental health care is stigma [11, 12]. Compared with other mental disorders (e.g., depression), the public’s attitudes towards psychosis are more negative, as psychotic disorders such as schizophrenia are often believed to be more unpredictable, more uncontrollable and more dangerous [11, 13–18]. Such negative attitudes may lead to discrimination and public rejection of people with psychotic disorders. Moreover, a person’s experiences of discrimination may in turn result in internalization of those stigmatizing beliefs (i.e., self-stigma), thereby exerting negative effects on their self-esteem, quality of life, social relationships and hope for recovery [19–25]. In addition, in a family-oriented society like China, it is taken for granted that family members should take care of and protect each other [26–30]. Hence, when a person is diagnosed with a mental disorder, the general public often attribute the onset of the illness to his/her family members’ negligence and incompetence [30–33]. Indeed, research has consistently shown that family caregivers of people with mental disorders, especially of those with psychotic disorders, often encounter discrimination and stigma [19, 33, 34]. In order to avoid being discriminated against, people with psychotic disorders and their family members may be inclined to conceal the illness or be reluctant to seek help [12, 17, 25, 34–38].

Poor mental health literacy may also have contributed to Chinese people’s underutilization of mental health services. Although psychotic disorders have long been the major priority for mental health care in China (e.g., the 686 program) [39], a meta-analysis of recent empirical studies found a low recognition rate of 18.4% for schizophrenia among the general public. This review also showed that fewer than 30% of people were willing to recommend a mental health professional for those with schizophrenia [40]. Improving mental health literacy and reducing stigma may play a role in improving rates of service use in people with psychosis. Improving the capacity of people in the social network of a person with psychosis to offer help is one aspect of this. This help is known as mental health first aid, which has been defined as “the help offered to a person developing a mental health problem, experiencing a worsening of an existing mental health problem or in a mental health crisis. The first aid is given until appropriate professional help is received or until the crisis resolves” ([41], p. 12).

As with physical first aid, mental health first aid is often provided by a member of the general public, such as a person’s family member, friend or colleague. Mental Health First Aid (MHFA) training is a 12-h training course that teaches members of the public how to recognize the symptoms of different mental disorders, how to provide initial help and how to assist a person with a mental health problem to get professional help [41]. MHFA training has spread to many high-income countries (e.g., England, US and Singapore) and a few low- or middle-income countries (e.g., Nepal, Pakistan and Bangladesh) [41]. Specifically, it has been shown to be effective in enhancing Hong Kong Chinese and Chinese-speaking Australians’ knowledge of mental health problems, improving their confidence to provide help, and reducing stigmatizing attitudes towards people with mental illness [42, 43]. However, these findings may not necessarily generalize to mainland China due to cultural and health care system differences.

The content of the MHFA training course is based on a series of guidelines developed using the Delphi expert consensus method [44]. These guidelines consist of a range of first aid actions (which have been rated as important or essential by expert panels of mental health professionals, consumers and caregivers) that a person can take to help someone with a mental illness [45]. In addition to informing the content of the training, the guidelines are also available on the MHFA website for the public to access (https://mhfa.com.au/mental-health-first-aid-guidelines). An evaluation of the effectiveness of the guidelines found that users who downloaded the guidelines improved in knowledge, skills and confidence in assisting a person with a mental health problem [45].

However, as the training course and the guidelines have been mainly developed for high-income countries, they may not be applicable for low- and middle-income countries with different health care systems and cultures, including mainland China. Previous Delphi studies, which focused on developing suicide first aid guidelines for Asian countries, namely Japan (high-income country), Philippines (lower middle-income country), India (lower middle-income country), Sri Lanka (upper middle-income country) and mainland China (upper middle-income country), have indeed identified some differences across cultures [46–51]. For example, considering a suicidal person’s religious and spiritual beliefs appeared to be more important when providing mental health first aid for Filipinos than for Japanese, Indian, Sri Lankan and Chinese. Additionally, compared with mental health professionals from the other countries, Japanese professionals were less likely to endorse the actions relating to dissuading the person from suicide, while the importance of family and friends was highlighted by Chinese panelists [46, 47, 50, 51]. Therefore, this study aimed to use the Delphi methodology to develop guidelines for members of the public providing first aid to people with psychosis in mainland China [51].

## Methods

The current Delphi study was conducted in four steps: survey development, recruitment of expert panel members, collection and analysis of data, and development of the final Chinese-language guidelines.

### Survey development

To ensure the guidelines are current and reflect best practice, re-development of mental health first aid guidelines is carried out every 10 years (e.g., the re-development of suicide and depression first aid guidelines) [52, 53]. Given the first English-language version of the psychosis guidelines was produced in 2008, new guidelines have recently been developed [54]. Items included in the current Chinese-language survey are the statements that received an “essential” or “important” rating from at least 78% of participants from at least one panel of experts (i.e., mental health professionals, caregivers or consumers) after the Round 1 English-language questionnaire used in the 2018 re-development [55]. The cutoff of 78% was chosen to minimize the risk of missing important items, due to the availability of results from Round 1 of the English-language survey (rather than the final results). It reflects our previous experience that items receiving an endorsement rating above 78% are highly likely to be endorsed for inclusion in the final guidelines after being re-rated in the subsequent round of survey. The items were carefully translated into Mandarin Chinese by the first author (WL) who is fluent in both English and Chinese. To ensure the accuracy of the translated items, the Chinese-language items were then back translated to English using Google Translate™ and checked by the first author. The Chinese-language survey was then checked and culturally tailored by the Chinese working group (WL, YH, YW and SL) who are not only familiar with MHFA training, but also have good knowledge of mental health treatment and health policies in China.

A total of 200 items were included in the first round of the survey. These items were classified into the following 13 sections: (1) Recognizing and acknowledging that someone may be developing psychosis (27 items); (2) Approaching the person (8 items); (3) Communicating with the person in a non-crisis situation (10 items); (4) Talking with the person in a non-crisis situation (18 items); (5) What to do with communication difficulties (9 items); (6) Being supportive (20 items); (7) How to help a person with postnatal psychosis (3 items); (8) Encouraging professional help in a non-crisis situation (12 items); (9) What to do if the person does not want professional help in a non-crisis situation (12 items); (10) How to help a person with hallucination and/or delusion in a non-crisis situation (14 items); (11) Assessing whether the person is in a crisis (3 items); (12) What to do if the person is in a crisis (in a severe psychotic state or behaving aggressively) (59 items); and (13) Self-care for the first aider (5 items). Participants were asked to rate each item on a 5-point Likert scale (1 = very important, 2 = important, 3 = unsure, 4 = unimportant, 5 = very unimportant) according to how important they believed it was for inclusion in the guidelines for a member of the public providing mental health first aid to a person with psychosis in mainland China. It should be noted that in order to shorten the length of the survey, two subsections with different situations but same items in Section 12 of the English-language survey – “what to do if the person is *in a severe psychotic state*” and “what to do if the person *behaves aggressively*” – were combined and presented as “what to do if the person is *in a crisis*” in the Chinese-language survey for 54 items. The survey was administered online via a Chinese online survey website, Questionnaire Star. Paper surveys were also used if access to the web survey was not possible.

### Expert panel recruitment

Potential participants from mainland China were identified and invited by YH and YW to join one of two expert panels: professional or lived experience. To be considered eligible, participants had to meet the following criteria:

1. 18 years of age or older;
2. Secondary school education or above;
3. Professional panel – Have at least 2 years’ working experience as a mental health professional or researcher in the field of psychosis;
  Lived experience panel – Have a lived experience of psychosis and feel well enough to participate, or have experience in caring for someone with psychosis.

Anonymity of panel members were ensured as the first author (WL), who was not involved in the participant recruitment procedure, was the only one who had access to the survey responses. Personal information provided by participants including their names (or pen names) and email addresses was deleted from exported data prior to analyses. Online (or paper) consent to participate was obtained before participants complete the survey via choosing “I consent to proceed” option.

### Data collection and analysis

Data were collected over two rounds of a survey between June and November 2019. Items were accepted for inclusion in the final guidelines if at least 80% of participants from each of the panels rated them as “very important” or “important”. Items were re-rated in the subsequent round of the survey if they were rated as “very important” or “important” by 70–79.9% of participants from at least one panel. Items were excluded if they were rated as “very important” or “important” by less than 70% of participants from at least one panel. After the first round of the survey, participants received a summary of the results including the number of endorsed items, a list of excluded items and a list of items that needed to be re-rated in the second round, together with each panel’s ratings for the excluded and re-rated items.

In addition to the 200 items derived from the results of the Round 1 English-language survey, the Round 1 Chinese survey also included open-ended questions at the end of each section asking participants to provide feedback and suggest helping actions that were not covered in the current questionnaire but important for members of the public carrying out mental health first aid in mainland China. The comments were carefully reviewed by WL, NR and YH, and new items were generated based on suggestions that contained new actions. The Round 2 survey comprised these new items and any items needing to be re-rated. Items that received comments on the expression of language were re-phrased and included in the second round as well.

### Guidelines development

The first author incorporated and wrote the endorsed items into a guidelines document, and the document was then sent to the Chinese working group for comments on the structure and wording. The final draft of the guidelines was also disseminated to participants (i.e., expert panel members) for suggestions and feedback. The final guidelines can be found in the online supplementary material.

### Ethics approval

This study was approved by the University of Melbourne Human Ethnics Committee (HREC No.1750853.1) and the Shanghai Mental Health Center Human Ethics Committee (IRB No.2018–62).

## Results

### Round 1

A total of 76 experts were invited to complete the Round 1 survey. Four of them who completed the survey in less than 10 min were excluded from the final data analysis due to concern about response quality. The professional panel (*N* = 31) comprised 25 psychiatrists, 3 public health physicians specializing in mental health, 1 mental health nurse, 1 psychotherapist and 1 community mental health practitioner. The average age was 45.5 years (*SD* = 9.8, range 28–66), with 16 males (51.6%) and 15 females (48.4%). Most of the panel members were from Shanghai (*N* = 15), 4 were from Beijing, 2 were from Zhejiang, with 1 each from Guangdong, Guangxi, Henan, Hubei, Hunan, Jiangsu, Liaoning, Shandong, Shaanxi, and Sichuan. The lived experience panel (*N* = 41) included 30 family members of people with psychosis and 11 non-mental health professionals who had experiences for caring someone with psychosis. Out of these 41 panel members, 11 were male (26.8%) and 30 were female (73.2%). The average age was 57.2 years (*SD* = 15.2, range 23–77). All participants were from Shanghai. It should be noted that despite our efforts, it was difficult to recruit consumers who were eligible to participant.

**Fig. 1:**
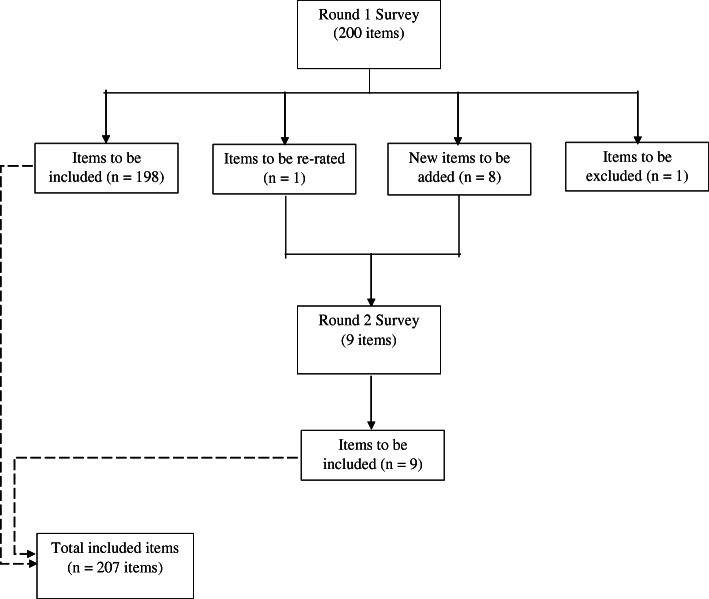
The number of items that were included, re-rated and excluded at each round of the survey

Two hundred items were rated in Round 1, and a total of 198 were endorsed for inclusion in the guidelines, 1 was rejected and 1 entered the Round 2 survey for re-rating (Fig. 1). A list of the endorsed, rejected and re-rated items can be found in the online supplementary material. The endorsement rates from the professional panel and the lived experience panel were significantly correlated (*r* = .44, *p* < .001), but some differences were also identified. As per previous research [53], items that were rejected by one panel but endorsed by the other, and with a difference of ≥10% on the ratings are listed below:

- “The first aider should take into consideration the spiritual and cultural context of the person’s behaviours” (professional: 94%; lived experience: 78%);
- “The first aider should not tell the person to get their act together” (professional: 58%; lived experience: 82%).

The endorsement ratings were also compared across the Chinese and English-language panels (i.e., Chinese professional panel versus English-speaking professional panel; Chinese lived experience panel versus English-speaking lived experience panel). It should be noted that comparison was not made between the Chinese and English-language panels on 54 items from section 12 – “What to do if the person is in a crisis (in a severe psychotic state or behaving aggressively)” – as the section had been revised by the Chinese working group. As shown in Table 1, notable differences were found in 10 items relating to knowledge about psychosis, effective communication, social support and getting professional help. In Round 1, participants were also encouraged to provide feedback and suggest new items that were not covered in the questionnaire. Accordingly, a total of 48 comments were received from both panels and 8 new items were created based on the suggestions with new helping actions (Table 2).

**Table 1:** Items with notable differences on the endorsement ratings between the Chinese expert panel and the English-language expert panel

Items	Rating			
	Professional		Lived experience	
	Chinese	English-language	Chinese	English-language
*Section 1. Recognizing and acknowledging that someone may be developing psychosis*				
The first aider should be aware that psychosis is not contagious.	97%	74%	–	–
The first aider should be aware that psychosis is not an intellectual disability.	97%	78%	–	–
*Section 2. Approaching the person*				
The first aider should approach the person face-to-face, if possible.	90%	78%	88%	75%
*Section 3. Communication (in a non-crisis situation)*				
The first aider should avoid using psychiatric terms when talking to the person.	100%	74%	97%	75%
*Section 5. Communication difficulties*				
If appropriate and feasible, the first aider should check with others who know the person for advice on the best way to communicate with them.	100%	74%	–	–
*Section 6. Being supportive*				
The first aider should not tell the person to get their act together.	58%	83%	–	–
The first aider should try to determine whether the person has a supportive social network and if they do, the first aider should encourage them to use these supports.	100%	74%	–	–
*Section 8. Encouraging professional help (in a non-crisis situation)*				
When encouraging the person to seek professional help, the first aider should focus on particular symptoms that are concerning the person and how treatment may help.	87%	70%	97%	75%
The first aider should provide the person with a range of options for seeking professional help.	–	–	97%	75%
*Section 10. Hallucination and delusion (in a non-crisis situation)*				
If the person is experiencing paranoia, the first aider should give the person simple directions, if needed, e.g. “sit down, and let’s talk about it”.	97%	78%	–	–

*Note.* items with notable differences are those which were rejected by one panel but endorsed by the other, and with a difference of ≥10% on the endorsement ratings

**Table 2:** New items generated from the comments with novel ideas

Comments	New items
*Section 2. Approaching the person*	
(the first aider should) contact the person’s family arriving at the scene immediately	The first aider should contact the person’s family immediately.
*Section 3. Communication (in a non-crisis situation)*	
make an appointment and get to know the situation in advance	The first aider should arrange a time to meet with the person in advance so they can have time to find out information about the person’s situation.
(the first aider should) check the person’s current physical condition first, such as whether he/she is thirsty, or feeling uncomfortable	The first aider should ask the person if they have any immediate physical needs, e.g. for food or water.
*Section 7. Postnatal psychosis*	
(the first aider should) provide some information about postnatal psychosis to the person’s family	The first aider should provide information about postnatal psychosis to the person’s family.
For the item “if a mother has delusions that involve her baby, the first aider should call a mental health crisis team immediately”, a clear definition of “mental health crisis team” should be provided, and make sure that the service is available in China	If the first aider thinks a mother may be experiencing postnatal psychosis or has delusions that involve her baby, they should call emergency services immediately, as it can escalate rapidly and delays in treatment can lead to increased risk for the mother and her baby.
*Section 8. Encouraging professional help (in a non-crisis situation)*	
For the item “the first aider should ask the person whether they have a doctor they trust, and if they do, the first aider should encourage them to seek professional help from their doctor”, there may be other professionals that the person trusts, such as a psychotherapist, a counselor etc.	The first aider should ask the person whether they have a health professional that they trust (e.g., a psychotherapist or counselor), and if they do, the first aider should encourage them to seek professional help from that professional.
To understand the person’s medical history and interpersonal relationships, can the first aider communicate with the residents’ committee in where the person lives or others who know the person well?	If possible, the first aider should seek information about the person (e.g., medical history, interpersonal relationships) from the residents’ committees or others who know the person well.
*Section 12. When the person is in crisis (in a severe psychotic state OR behaving aggressively)*	
If possible, (the first aider should) find out if the person has a history of aggression through reliable sources immediately	If possible, the first aider should find out if the person has a history of aggression.

### Round 2

The Round 2 survey consisted of the 8 new items and the one needing to be re-rated (Fig. 1). A total of 29 professionals (Male = 51.7%; *M*_age_ = 46.4, *SD* = 9.4, range 31–66) and 20 lived experience panelists (Male = 30%; *M*_age_ = 51.5, *SD* = 15.9, range 23–70) completed the questionnaire. All of the 9 items were endorsed, therefore another round of survey (i.e., Round 3) was not conducted. A sum total of 207 endorsed items from the two rounds of the survey formed the basis of the Chinese-language psychosis first aid guidelines.

## Discussion

The current Delphi study aimed to develop guidelines for members of the public providing mental health first aid to people with psychosis in mainland China. A total of 207 statements were endorsed by both professional and lived experience panels and were included in the guidelines. These guidelines involve a variety of strategies that a person can use when providing assistance, including how to recognize the signs of psychosis, how to approach someone who may be developing psychosis, how to assess whether the person is in crisis, what to do if the person is in crisis, how to communicate with the person, what to do with communication difficulties, how to support the person, how to help someone with postnatal psychosis, how to encourage professional help, what to do if the person does not want professional help, how to help a person with hallucinations or delusions, and self-care strategies for the first aider. The resulting guidelines will be available for dissemination as a standalone guide and will also inform the development of the Chinese MHFA training course.

Differences between the professional and lived experience panels were noted when examining specific items. There were two items which did not reach consensus to be included in the guidelines after the first round of the survey. The item “the first aider should take into consideration the spiritual and cultural context of the person’s behaviors” was highly endorsed by the professional panel after both rounds of the survey (Round 1: 94%; Round 2: 97%) but was not endorsed by the lived experience panel after the Round 1 survey (78%) and only received an endorsement rating of 80% after being re-rated in the second round. This difference across panels may be attributable to the geographic regions in which our respondents were recruited. Members in the professional panel were recruited across several regions in mainland China, including some provinces which contain a large proportion of China’s ethnic minorities such as Guangxi. Hence, the professional panel members may have a better understanding of the cultural influence on a person’s behavior, in comparison with the lived experience panel members who were all from Shanghai. In addition, while 82% of lived experience panel members endorsed that “the first aider should not tell the person to get their act together”, only 58% of professionals rated this statement as either very important or important. This item was rejected by the professional panel may be because it does not include specific support strategies. Indeed, one professional panel member commented that concrete details of available support services that the first aider can use should be provided.

Importantly, there were also a number of differences between the Chinese and English-language expert panels on their endorsement ratings for several statements [54]. First, although 83% of English-speaking mental health professionals endorsed that “the first aider should not tell the person to get their act together”, only 58% of Chinese professionals believed this item as either very important or important for inclusion in the Chinese-language guidelines. This disagreement may reflect the way the item was translated. The translated Chinese phrase “*振作起来*” (*zhen zuo qi lai*) may fail to express the negative connotation associated with the original English phrase “get your act together”. Second, the Chinese expert panels gave higher endorsement ratings to statements relating to general knowledge about psychosis and how to support and communicate with someone with psychosis. For example, 100% of professionals and 97% of lived experience panelists from mainland China endorsed the item “the first aider should avoid using psychiatric terms when talking to the person”, whereas only 74 and 75% of English-speaking professionals and carers considered this statement as either essential or important for inclusion in the guidelines, respectively. The Chinese panel members’ higher endorsement rates may reflect their concern about the lack of mental health knowledge in the Chinese public. A recent systematic review has indeed demonstrated the Chinese public’s poor mental health literacy in terms of their ability to recognize different mental disorders and their knowledge and beliefs about available treatment [40].

Third, compared with their English-speaking counterparts, Chinese experts tended to emphasize the actions that involve the person’s family members or other social networks. These include “the first aider should try to determine whether the person has a supportive social network and if they do, the first aider should encourage them to use these supports”, “the first aider should try to involve the mother’s partner or family in minimizing any risk to the mother or baby” and “the first aider should be aware of the influence that the person’s family may have, e.g. the family may encourage or discourage the person from obtaining the care that they need”. Also, two new relevant items – “the first aider should contact the person’s family immediately” and “the first aider should provide information about postnatal psychosis to the person’s family” – were suggested and endorsed. These results again reflect the central role of family in caring for someone with psychosis in China. Furthermore, gathering information about the person’s situation was also emphasized by the Chinese panel members, including a highly endorsed item “if appropriate and feasible, the first aider should check with others who know the person for advice on the best way to communicate with them”, and three new items “the first aider should arrange a time to meet with the person in advance so they can have time to find out information about the person’s situation”, “if possible, the first aider should seek information about the person (e.g., medical history, interpersonal relationships) from the neighborhood committees (*居委会 ju wei hui*) or others who know the person well” and “if possible, the first aider should find out if the person has a history of aggression”. These actions would ensure the first aiders are capable to make informed decisions about what should be done.

Fourth, the high endorsement rates for the items under the sections “encouraging professional help” and “what to do if the person does not want professional help” (professional: 97–100%; lived experience: 87–100%) may imply that the first aider’s support role in encouraging a person to seek professional help may be more important when providing mental health first aid in mainland China. Indeed, systematic reviews of Chinese mental health professionals’, patients’ and caregivers’ mental health literacy found that while more than 80% of professionals and caregivers believed that people with mental health problems should seek professional help, only 33% of patients agreed that professional services should be sought (Li & Reavley: patients’ and caregivers’ knowledge and beliefs about mental illness in mainland China: a systematic review, submitted; Li & Reavley: the mental health literacy of healthcare workers in mainland China: a systematic review, submitted). The differences between Chinese and English-language expert panels on the endorsement ratings may be partly due to the different composition of the panels. For example, while the Chinese professional panel mainly composed psychiatrists, the English-language panel comprised a diverse range of mental health professionals from different high-income countries (e.g., Australia, Canada, UK) were involved in [55].

The current results need to be considered in the context of the following limitations. Firstly, it was impossible to recruit some types of clinicians, such as clinical psychologists and occupational psychologists, due to the lack of these mental health professionals in mainland China [56]. In addition, consumers were not involved in the current Delphi study as it was difficult to approach people who were unwell from hospital settings or to obtain consent from their guardians, and the absence of consumer advocacy groups similar to those in high-income countries [55]. Future research using one-to-one interviews with a small group of consumers may provide some initial understanding of what they think about providing mental health first aid for psychosis in China. Secondly, the lived experience panel members were all recruited from Shanghai, and this may limit the generalizability of the current findings to other regions, given mental health resources are unequally distributed across different areas in China [57, 58]. Furthermore, it should be noted that 28 lived experience panelists completed the first round of the survey using paper-and-pencil, and missing values were observed in 25 returned questionnaires. However, as we recruited a total of 41 lived experience panelists, this guaranteed that there were at least 30 respondents for each survey item [59]. Thirdly, a high dropout rate of lived experience panel members was noticed – from 41 in the first round to 20 in the second. It could be that the time commitment needed for finishing the Round 1 survey (i.e., approximately 1 h) may have deterred the participants from completing the subsequent questionnaire. Importantly, the minimum requirement of 20 members per panel was met [59].

## Conclusions

This study used the Delphi consensus method to produce culturally appropriate psychosis first aid guidelines for members of the public in mainland China. Gaining more knowledge about psychosis and gathering information about the person’s current status before providing assistance were emphasized by both professional and lived experience panels. In addition, compared with their English-speaking counterparts, the important role of the person’s family members in providing first aid was also highlighted by the Chinese panelists.

## Supplementary information

**Additional file 1.**

**Additional file 2.**

## Data Availability

The dataset used and analyzed during the current study are available from the corresponding author on reasonable request.

## Abbreviations

DUP: Duration of Untreated Psychosis
MHFA: Mental Health First Aid
WHO: World Health Organization
