# Supplementary material for: Development of Chinese mental health first aid guidelines for psychosis: a Delphi expert consensus study
Source: BMC Psychiatry. 2020 Sep 10;20:443. doi: 10.1186/s12888-020-02840-5 (PMC7488132; doi:10.1186/s12888-020-02840-5)
Supplement: Supplementary file 2 — Additional file 2. [file 12888_2020_2840_MOESM2_ESM.docx]

# 精神健康急救指南 – 精神病篇

## 指南的目的

指南旨在指导公众如何为可能正在经历精神病问题的人（在本指南中称为“救助对象”或“对方”）提供初步帮助，即实施“精神健康急救”。

| 精神健康急救 (Mental Health First Aid, MHFA)：为发生精神健康问题，或现有精神健康问题恶化，或正在经历精神健康危机(如自杀或创伤经历)的人提供的**初步帮助**，直至对方获得适当的专业帮助或危机解除。 |
| --- |

所谓精神健康急救人员（后简称“急救人员”），即为经历精神病问题的人提供救助的非精神卫生专业人士，他们可以是救助对象的家人、朋友、同事或邻居，等等。急救人员的作用是为经历精神病问题的人提供初步帮助，直至对方获得适当的专业帮助或危机解除。

## 指南的制定

指南的内容是在综合经验丰富的精神健康领域专家和有照顾精神病患者经验人士意见的基础上制定的。

## 指南的使用

指南所提供的指导方针仅为一般性建议。每个救助对象的情况都是不同的，急救人员需要根据对方的情况对所提供的帮助作出适当调整。

## 什么是精神病

在本指南中，精神病泛指使人脱离现实且伴有妄想或幻觉的精神疾病，如精神分裂症。精神病会对患者的日常生活、人际关系、工作或学习造成巨大影响，并且会使其自身和身边的人非常痛苦和混乱。精神病症状（如幻觉、妄想）也会出现在其他精神疾病中，如双相障碍、成瘾物质所致精神障碍等。患者可能会经历多次精神病发作，但每次发病的间隔期会处于健康状态。精神分裂症或其他精神病**并非**“人格分裂”或智力障碍，更不会传染。

值得注意的是，并非所有患有精神病的人都会认为自己的精神病症状是一个问题，因为其精神病可能还没有对他的生活和工作造成负面影响或使其处于危机状态，比如并没有出现严重的精神病状态，没有自杀想法或行为，也没有出现攻击行为。

该指南旨在指导非精神卫生专业人员为那些受到精神病负面影响的人提供精神健康急救。除另有注明外，本指南中所提供的信息适用于非危机情况下实施精神病紧急救助。

## 如何识别并确认某人可能患有精神病

对精神病的早期征兆和症状识别是非常重要的，即使对方的症状并不是非常明显，也不能忽视，因为精神病的症状可能会因人而异或随时间推移逐渐显现。作为急救人员，你应该对一系列可能会引发精神病的因素有所了解，如极度压力、创伤或药物滥用等。精神病的症状可能与使用药物或酒精有关，但不要以为对方所表现出的症状就一定是误用药物或其他成瘾物质所导致的，也不能简单推断这一症状只是“正常的阶段性的人生起伏”，更不应假设这些症状会自行消失。另外，救助对象的异常变化可能正是患精神病的早期征兆，所以救助者应避免把一个年轻人的功能变化或反复出现的古怪行为看成是阶段性行为或青春期行为。同时，也应考虑对方行为举止背后的信仰和文化背景。救助对象正在经历的精神病也可能是某一单次发作或是其他疾病（如双相障碍或痴呆症）的一部分。如果无法确定其是否患有精神病，应向精神卫生专业人员寻求专业帮助。

## 如何接近救助对象

患有精神病的人可能不会主动寻求帮助。如果你担心某人可能患病，应尝试在一个尽可能安全、舒适且无干扰的环境中，以关怀、无偏见的方式，与其面对面接触。不要以挑衅的方式接近对方。你需要预留出足够的与对方交谈的时间，这样才不会显得仓促。在与对方交谈时，不管其情绪状态如何，你都应该尽量保持冷静，同时根据对方的行为方式适当地调整自己的做法和交流方式。例如，如果对方多疑且躲避眼神接触，你应该敏锐地察觉并给予对方必要的空间。如有必要，你应及时通知其家属到场，因为社会支持对救助对象是有帮助的。但你也要意识到，对方很有可能会缺乏社会支持，因为在日常，他可能会孤立自己或者其行为会使别人远离他。

## 如何在非危机情况下与救助对象沟通

### 如何与对方交谈

救助对象可能知道发生了什么，也可能完全没意识到，或因困惑、恐惧否认自己已经患病。你可以考虑提前安排合适的时间与其或其家属见面，以确保有充足的时间可以了解对方目前的情况。在交谈初期，你应该首先询问对方是否愿意谈论他的感受，也可以先寻找一些共同话题与其进行讨论，然后逐步提出与疾病相关的具体问题。如果对方已经注意到自身行为的变化，你可以询问其是否对这些变化感到苦恼，并允许他谈论自己的经历、感受和信仰。但你要知道，对方在描述自身症状时可能会含糊不清，并且可能会强调只是躯体症状而不是精神疾病症状。在叙述过程中，他也有可能会被自己的想法和感觉惊吓到。如果奇因自身经历而感到忧虑，你可以询问怎样可以使他感到安全或可控。在谈论他的未来时，应避免表露出消极或悲观的态度。

如果对方否认自己有问题或不愿谈论正在经历的事情，你不要与其争论，也不要试图去改变他的想法，而是应该专注于倾听并询问对方，自己可以做点什么来帮助他，并且要让他知道，以后也可以找自己交谈。切记，不要向对方推测诊断结果。

| **与救助对象沟通时的“应该”与“不应该”** 与救助对象交流时，你**应该**：   - 表达出同理心 - 使用日常用语（如“压力”）或用对方自己的语言来表述，以使对方的经历正常化 - 不带评判性地倾听对方讲话 - 仔细聆听对方讲话并对所听到的内容做出反应，让对方了解你正在听并理解正确 - 认可对方所说的事情，对其感受表示理解，如“这听起来确实很令人沮丧”或者“听起来好像你不知道该怎么做” - 尽量让对方把握互动的节奏和方式 - 尽量减少不安或紧张的肢体语言，如抖腿、坐立不安或咬指甲   与救助对象交流时，你**不应该**：   - 使用精神病学术语 - 使用可能会使对方感到被冒犯的侮辱性词语，如“疯了”“疯子”“神经病” - 站着或在对方身旁徘徊，如果对方是坐着的 |
| --- |

### 如何解决沟通中遇到的困难

对方可能无法正常交流（如答非所问，或从一个话题转移到另一个话题），也可能会缺乏非语言信息（如面部表情和语调），或者需要多次尝试对话后才能开口说话，你应该给予他充足的时间来回答问题或表达，因为他可能有信息处理困难，或者有理解和思考困难。要知道，即使对方的回应是有限的，但这并不代表他听不懂你在说什么；如果对方只表现出有限的情绪，也不代表这是他的全部感受。

尝试用清晰简单的沟通方法，避免使用复杂的语言（如隐喻或讽刺），必要时复述对方所说的内容。如果适当可行的话，你也可以咨询知情人，了解与其沟通的最佳方式。在交谈过程中，对方可能会做出不符合对话情景的情绪反应 （如大笑），也可能会出现幻听，这会使沟通变得困难。如果对话变得紧张或激动，应该稍事休息，让自己和对方恢复平静。如果对方出现令人讨厌或愤怒的行为，应给予理解，要知道他正在经历自身所无法控制的病痛折磨，在这种情况下双方都有可能会感到痛苦，但不应责怪他，或认为他的行为是针对自己的。

### 如何表示支持与理解

一方面，询问对方是否希望得到帮助，以及希望得到怎样的帮助。另一方面，要表达你愿意为他提供帮助并能够为他提供哪些帮助，并表达出想要确保他安全的诚意。比如，是否需要为他安排儿童托管或带他去看医生之类的实际帮助。同时，询问他是否因为某些压力引发了他的症状，以前是否有过类似经历，如果有，他采取了哪些有用的措施。

尝试了解对方是否具有社会支持网络，如果有，你应该鼓励他使用这些支持。如果觉得合适，可以询问对方，你能否经常与其联系。如果你一直与救助对象保持联系，那么需要注意对方是否有症状恶化的迹象。如果对方是青少年，当他与父母或其他人联系、交谈的时候，你应提出陪在其身边。

你应该始终尊重救助对象，要理解他可能不会遵循你提出的任何建议。在试图改变他的行为时，不要用后果来威胁他。应该认可他能够与你交谈的勇气，并告诉他，自己理解他可能被所发生的事情吓到了。在互动时，救助者应该避免居高临下或轻描淡写的表述方式，例如，“打起精神来”，“我相信会过去的”，或“情况本可能会更糟”。也不应轻视或嘲笑对方，即使他所说的话毫无意义。如果有其他人在场，不应旁若无人地谈论救助对象。切记，除非救助对象对自身或他人构成威胁，否则必须尊重他的隐私权，并给予保密。

### 如何应对妄想与幻觉

| 什么是妄想和幻觉 妄想是一种错误的信念，例如，在毫无事实根据的情况下，坚信有人要伤害自己或坚信自己有特殊使命。  幻觉是一种虚假的感觉，常出现在听觉、视觉等方面。 |
| --- |

首先你要知道妄想或幻觉对救助对象来说是非常真实的。对方所经历的妄想或幻觉可能会导致其不相信别人，哪怕是亲人。如果对方想要谈论他的妄想或幻觉，你应该倾听以示同理心，以便更好地理解对方正在经历的事情。与此同时，你应该试着理解和同情对方对自身信念和经历的感受，而不是对这些信念和经历的内容做出评判。如果对方出现幻听，他可能会通过自言自语等行为方式，以回应自己听到的声音。

| 在应对妄想与幻觉时不应做的事情：  - 假装认同对方的妄想或幻觉 - 忽视、轻视或与对方争论他的妄想或幻觉 - 对对方的妄想或幻觉表现出惊慌或局促不安 - 取笑对方的妄想或幻觉 |
| --- |

如果对方表现出猜疑、偏执，要避免鼓励或激化这一情况，如，与其窃窃私语或私下谈论他的猜疑。如果对方已处于偏执状态，应在需要的情况下，给他简单的指示，如，“让我们坐下来谈一谈”。应尽量陪伴在他身边，但要保持一个让双方都舒适的距离。在行动之前告知对方自己要做什么，比如需要拿出手机，避免出现可能会加重对方猜疑的举动。在确保自身安全的前提下，应尝试鼓励并支持对方远离使其恐惧的事物。

## 鼓励救助对象寻求专业帮助

在精神病发现早期进行治疗是最有效的。如果可能，你可以与对方所在当地村（居）委会或对其比较了解的人员沟通，以及时了解对方的情况（如，病史、人际关系）。在鼓励对方寻求专业帮助时，应该把焦点放在对方担心的特定症状上，思考怎样的治疗可以帮到他，不应使用威胁或对抗的方式。要告诉对方，他可以获得帮助，并且让他明白，适当的专业帮助可以改善他目前的状况，让他看到希望。同时不要忽视家人对他的影响，比如，鼓励或阻止他寻求帮助。由于精神病的症状可能是身体疾病引起的，你应该鼓励对方进行体检。你要熟悉当地有哪些可用的服务资源，了解可以获得专业帮助的途径有哪些，以供对方选择，例如通过社区医生转诊到专科医生。询问对方是否有信任的精神卫生专业人员（如精神科医生、心理治疗师、心理咨询师等），如果有，你应该鼓励他向该专业人员寻求帮助。作为急救人员，对有助于精神病治疗的方法你也应有一些基本的了解。

### 如果对方不想寻求专业帮助

你应该首先关心对方此时此刻的身体状况，并弄清楚他当下的迫切需求是什么（如食物、水）。如果对方不愿寻求专业帮助，要保持耐心，因为患有精神病的人通常需要一定的时间才能认识到自己确实是生病了。在他没有认识到自己生病时，可能会十分抗拒别人鼓励他寻求帮助。救助者要平静地向对方表达自己的担心，让他明白如果不寻求帮助可能带来的潜在影响。要做好足够的心理准备，在对方愿意寻求专业帮助前可能需要与其进行多次对话。

在鼓励对方寻求帮助时，要尝试引导对方寻找那些他同样认为是问题的症状，然后建议他有针对性地为解决这个症状而去寻求帮助。例如，如果对方说他在别人面前感到焦虑，你就应该鼓励他为缓解焦虑而去寻求帮助。要强调获得帮助的潜在好处（如缓解焦虑或恐惧症状）。向对方保证他是可以去寻求帮助的，并指出这是有勇气的行为，而不是软弱或失败的象征。如果对方不愿寻求专业帮助是因为害怕别人想要伤害他，你应该鼓励他与信任的人谈论自己正在经历的事情。也可以给对方提供其所在社区的其他帮助资源，如家访或社区服务。尽量与对方保持良好的关系，因为他未来可能会希望得到你更多的帮助。切记，对方有权拒绝治疗，除非他符合强制治疗的标准。避免以强制治疗或住院来威胁他。

## 如何应对产后精神病

如果你认为一位母亲可能患有产后精神病或已出现与其孩子有关的妄想，则应立即给急救中心打电话并带她去精神专科就诊，因为病症可能会迅速升级，如果延误治疗会增加对母亲及孩子的危险。在确保对方得到专业帮助之前，尽量保证有人一直陪在她身旁。尽量让其伴侣或家人参与，并向他们提供一些产后精神病的知识，以把母亲或婴儿所面对的危险降到最低。

## 当救助对象处于严重发作或有攻击性的紧急情况时

### 如何评估对方是否处于危机之中

评估救助对象对自身及他人造成伤害的风险。虽然患有精神病的人通常不具有攻击性，并且比起伤害别人更可能伤害自己，但精神病的某些症状（如幻听或幻视）还是有可能导致其变得有攻击性。如果你怀疑对方可能会对自己或他人构成威胁，那么你应该立即与应急处理人员或部门联系，如社区干部、基层医疗卫生机构的精防人员、警察，必要时离开现场。如果你不认为对方存在立即受到伤害的风险，但仍担心他的人身安全，则应该询问对方是否有关系亲密的人可以帮助他以确保他的安全。如果你认为对方存在自杀的风险，应该参照“精神健康急救-自杀篇”指南来帮助对方。

### 如果对方处于危机中，如何应对

如果对方变得具有攻击性或处于严重精神病状态，你应该认真对待他的任何威胁或警告，尤其是在他认为自己正在受到迫害的时候，因为他可能会根据妄想或幻觉的内容行事。在接近对方时要小心谨慎，你的首要目标应该是尽力保护他、也保护自己以及身边其他人免受伤害。如果可能，立即通过有效渠道了解他过去是否有过攻击行为。允许对方表达自己的感受并告诉他自己会给予他帮助，不要以敌对、训诫、争辩或挑衅的方式应对。不要威胁对方，因为这可能会增加对方的恐惧或引发其攻击行为。不要进一步刺激对方，也不要用联系紧急服务来威胁对方。尽量满足对方的要求（除非这些要求是过分的或不安全的），因为这样做可以让对方在某种程度上获得控制感。如果对方事先有预防复发的方案，你应该遵循这些方案行事。

用简单的语句冷静地与对方交谈，不要对其大吼大叫或提高嗓音。询问对方自己可以做些什么来帮助他，但应避免问过多的问题，因为这可能会引发对方的抵触情绪，使他更加愤怒。试着找出对方是否有信赖的人（如朋友或家人），如果有，你应该尝试寻求他们的帮助。如果有其他人到达现场，你应该向他们说明自己的身份，并表明自己是来提供帮助的以及打算提供怎样的帮助。如果有多人在场，尝试给救助对象周围营造一定的空间，不让他感到拥挤，并鼓励一次只有一人讲话。不要与在场的任何其他人争论最佳行动方案。如果有与救援无关的人在场，应该让他们离开。如果你与救助对象单独在一起，又不能继续陪伴他时，应联系其他人过来，直到专业人员到达。

如果救助对象对自身安全有不切实际的恐惧，你应该向他保证他是安全的，并尝试找出能够让他感到安全和可控的方法或途径。询问他是否感到安全，如：“你看起来很忧虑，有什么我可以帮忙的吗？”“你觉得安全吗？还是你在害怕什么？”询问他是否希望减少干扰和刺激，例如，关掉电视、调弱屋内灯光等。

尽量与对方保持能够维持互动的安全距离。确保有一条畅通的通道通往出口。如果确实需要靠近对方或与对方有身体接触，你应该先询问并征得他的同意，例如：“你介意我坐在你旁边吗？” “你手臂受伤了，我可以用急救箱帮你包扎一下吗？”保持冷静，尽量减少自己的情绪外露，尽量不表现出恐惧或焦虑，避免紧张的行为（例如，抖腿、坐立不安、突然行动、说话过快）。如果感到害怕，应该立即寻求外界帮助，不要把自己置于危险之中。可以联系其他人来陪伴自己，直到专业人员到达。

在寻求专业帮助方面，尽量给对方有选择余地，这样可以让他有控制感，如“你是想让我陪你去医院还是希望小王陪你去”。尽可能让他立即接受精神卫生专业人员的评估。如果觉得自己亲自带他去医院不安全，应该联系警方和/或急救中心。如果你所联系的服务机构忽视了你的担忧，那么你应该坚持不懈地继续尝试，如打电话给其他服务机构。在请求精神科专业人员或警察援助时，不应直接将救助对象称为“精神病患者”，而是应简明扼要地说出自己观察到的所有症状、行为和眼前的问题；如果知道他曾被诊断患有精神病，则应直接说明。到医院后，如果你觉得与他的关系合适，应该直接把他的情况告诉医生。

尽量限制对方接触可以用来伤害自己或他人的东西。在保证安全的前提下，尝试从对方所处的环境中移除任何武器或可用作武器的东西。如果他携带武器或可用作武器的东西，你不应接近他而应立即报警。如果已与紧急服务取得联系，应让紧急服务人员了解救助对象是否携带武器，或其身边是否有可用作武器的东西；了解对方是否存在自杀或自残的风险。当紧急服务人员到达现场时，你应该尽量在第一时间与他们会面，这样你就可以在紧急服务人员接触救助对象之前向其解释当前的情况。

如果你无法使情况缓和，或救助对象的攻击行为已升级到失控的程度，你应该离开现场并打电话给紧急服务寻求专业协助（如警察）。需要注意的是，你所采取的某些行动（如报警）可能会加剧对方的攻击性。打报警电话时，你应该告诉警察，救助对象可能患有精神病，需要警察的协助来控制对方的攻击行为并获得医疗救助。

## 急救人员的自我调节

发现亲近的人患有精神病或帮助患有精神病的人，可能会让救助者自己产生震惊、慌乱、内疚、恐惧、悲伤、愤怒、挫败等情绪，这是常见的正常反应。你应该照顾好自己的心理和身体健康，尽量不要把对方说的话当作是针对自己的。如果感到自己所承担的角色有压力，则应在对救助对象信息保密的同时，为自己寻求帮助（例如，参加支持团体或组织、寻求心理健康专业人员或朋友的支持）。尝试一些自助策略来减轻压力（例如，放松练习、规律运动、睡觉、健康饮食）。不必要求自己能为救助对象解决所有问题，这只会徒增压力。
